# An open data repository for steady state analysis of a 100-node electricity distribution network with moderate connection of renewable energy sources

**DOI:** 10.1016/j.dib.2017.08.040

**Published:** 2017-09-05

**Authors:** Stavros Lazarou, Vasiliki Vita, Lambros Ekonomou

**Affiliations:** Department of Electrical and Electronic Engineering Educators, School of Pedagogical & Technological Education (ASPETE), Heraklion Attikis, 141 21 Athens, Greece

**Keywords:** Electrical power systems, Electric power distribution, Smart grid, Power system modelling and simulation, Steady State, Power flow, Matpower, Matlab, Modelling, Simulation

## Abstract

The data of this article represent a real electricity distribution network on twenty kilovolts (20 kV) at medium voltage level of the Hellenic electricity distribution system [1]. This network has been chosen as suitable for smart grid analysis. It demonstrates moderate penetration of renewable sources and it has capability in part of time for reverse power flows. It is suitable for studies of load aggregation, storage, demand response. It represents a rural line of fifty-five kilometres (55 km) total length, a typical length for this type. It serves forty-five (45) medium to low voltage transformers and twenty-four (24) connections to photovoltaic plants. The total installed load capacity is twelve mega-volt-ampere (12 MVA), however the maximum observed load is lower. The data are ready to perform load flow simulation on Matpower [2] for the maximum observed load power on the half production for renewables. The simulation results and processed data for creating the source code are also provided on the database available at http://dx.doi.org/10.7910/DVN/1I6MKU.

**Specifications Table**

**Table:** 

Subject area	*Electrical Engineering*
More specific subject area	*Power System analysis*
Type of data	*Processed data on.m format, tables*
How data was acquired	*The data represent a real part of the electricity grid.*
Data format	*MATPOWER, MATLAB format.m files, excel file*
Experimental factors	*The data are ready to perform loaf flow simulation for the maximum observed load power on the half production for connected renewables.*
Experimental features	*The data are based on Matpower format for load flow, power system analysis.*
Data source location	*The line on which these data are based is located in Greece*
Data accessibility	*The data are available on Harvard Dataverse*: http://dx.doi.org/10.7910/DVN/1I6MKU

**Value of the data**

• The source code represents a real distribution network of moderate but adequate size. It is ready to perform steady state analysis. The data are suitable for studies in load aggregation, storage, demand response and consequently for the optimal design of distribution grid's topology. Their format on Matlab [3] allows for quick and easy integration with emerging technologies and/or proven scientific methods.
• The fact that these data are based on a real distribution grid enhances the applicability of the research to be conducted. The dimensioning of the lines, loads and generators currently corresponds to an operational network. Consequently, the results of the upcoming research using these data have the prerequisites to be also technically suitable to be applied by a network operator.
• The bibliography provides a wide selection of open source data for power system applications. CIGRE [4] and IEEE [5], [6] networks are regularly used but they are focused to high voltage transmission lines even if they provide examples on the distribution. Smart grids are regularly deployed to distribution level and a wide selection of networks could contribute to their implementation. This dataset seeks to cover the gap providing only line data on this voltage level additionally to the data already available.
• Our open source data are available for performing steady state, load flow analysis of the given network. However, further enhancement in terms of line and generators data could facilitate short-circuit and dynamic calculations and consequently to support protection and stability studies. This is part of future work but further information is required to increase results accuracy.

## 1 Data

### 1.1 General information

On the technical level, smart grids as such are considered an advanced discipline. The scientific community and the industry have allocated resources in describing and solving the main challenges as far as the interconnection of several types of facilities to the distribution level are concerned. However, additional effort needs to be allocated in achieving homogeneity in conducting smart grid simulations. This approach requires taking into consideration the specifics of each system and requires the accessibility to real networks to perform the research. Several organisations offer open source data of electricity grids. CIGRE [4] and IEEE [5], [6] networks are the commonly used. There are also available datasets from EPRI [7] and PNNL [8]. Additional datasets are available to the bibliography.

### 1.2 Line description

In any case, the distribution lines will remain the backbone of the distribution that is not going to change. Based on this way of thinking, this manuscript provides open access to a real distribution network line with the following special characteristics. It represents a rural line of fifty-five kilometres (55 km) total length, of a typical length for this type. It serves forty-five (45) medium to low voltage transformers and twenty-four (24) connections to photovoltaic plants. The total installed load capacity is twelve mega-volt-ampere (12 MVA), however the maximum observed load is lower. The connection points are given according to Table 1. The names of the nodes have been changed from the original ones in order to meet MATLAB requirements. All the above are provided below and at the attached online dataset (http://dx.doi.org/10.7910/DVN/1I6MKU).

**Table 1:** Connection points of line branches and their characteristics.

*Name*	*From node*	*To node*	*Length*	*Construction Type*	*R* (Ω/km)	*L* (Ω/km)
Line2	28	31	0.25	ACSR95	0.215	0.334
Line7	28	p2	2.305	ACSR95	0.215	0.334
Line3	31	39	0.488	ACSR95	0.215	0.334
Line11~	39	39_18	0.945	ACSR35	1.071	0.393
Line4	45	39	0.505	ACSR95	0.215	0.334
Line5	45	49	0.333	ACSR95	0.215	0.334
Line53	45	45_1A	0.132	ACSR16	1.268	0.422
Line54~	49	49_9	0.473	ACSR16	1.268	0.422
Line6	49	61	1	ACSR95	0.215	0.334
Line30~	61	68	0.55	ACSR95	0.215	0.334
Line16~~~~~	68	68_3	0.179	ACSR95	0.215	0.334
Line26	68	S_FBA_259	0.068	ACSR35	1.071	0.393
Line32~	68	74	0.242	ACSR95	0.215	0.334
Line18~~~~	74	80	0.521	ACSR95	0.215	0.334
Line34	74	74_1A_4	0.363	ACSR16	1.268	0.422
Line35~	80	87	0.6	ACSR95	0.215	0.334
Line38	87	87_3	0.225	ACSR16	1.268	0.422
Line42~	87	89	0.197	ACSR95	0.215	0.334
Line18~~~~~	89	91	0.208	ACSR95	0.215	0.334
Line41	91	S_FBP_14	0.008	ACSR35	1.071	0.393
Line43~	91	92	0.006	ACSR95	0.215	0.334
Line44	92	95	0.334	ACSR95	0.215	0.334
Line51	92	92_8	0.785	ACSR35	1.071	0.393
Line14~	39_18	39_51	2.419	ACSR35	1.071	0.393
Line12	39_51	39_94	3.125	ACSR35	1.071	0.393
Line19~	39_51	39_51_3	0.258	ACSR35	1.071	0.393
Line23~	45_16A	45_1A	0.871	ACSR16	1.268	0.422
Line24~~	45_1A	45_1A_4	0.14	ACSR16	1.268	0.422
Line105	61_10	61_7	0.304	ACSR95	0.215	0.334
Line24~~~~~	61_10	61_10_6	0.311	ACSR16	1.268	0.422
Line8	61_10	61_11	0.108	ACSR95	0.215	0.334
Line9	61_11	61_15A	0.469	ACSR35	1.071	0.393
Line11	61_15A	61_15A_6	0.465	ACSR35	1.071	0.393
Line15	61_15A	61_22A	0.661	ACSR95	0.215	0.334
Line16	61_22A	S_FBI_69	0.5	ACSR35	1.071	0.393
Line17	61_22A	61_28	0.447	ACSR95	0.215	0.334
Line18	61_28_9	61_28	0.326	ACSR16	1.268	0.422
Line113	61_32	61_33	0.082	ACSR95	0.215	0.334
Line118	61_32	61_32_3	0.3	ACSR16	1.268	0.422
NODE12~~~~~~~	61_32	61_28	0.343	ACSR95	0.215	0.334
Line122	61_32_16	61_32_31	1.365	ACSR16	1.268	0.422
Line19	61_32_16	S_FBI_137	0.023	ACSR35	1.071	0.393
Line121	61_32_3	61_32_16	1.275	ACSR16	1.268	0.422
Line126	61_32_31	61_32_31_1	0.102	ACSR35	1.071	0.393
Line20	61_32_31	61_32_162	10.181	ACSR16	1.268	0.422
Line21	61_33	61_42A	0.777	ACSR35	1.071	0.393
Line22	61_42A	61_45	0.286	ACSR35	1.071	0.393
Line23	61_42A	61_42A_9	0.702	ACSR35	1.071	0.393
Line24	61_42A_9	S_HBSE_470	0.076	ACSR35	1.071	0.393
Line25	61_42A_9	S_HBSE_700	0.414	ACSR35	1.071	0.393
Line115	61_45	61_45_4A	0.483	ACSR35	1.071	0.393
Line116	61_45_4A	61_45_12	0.677	ACSR35	1.071	0.393
Line24~~~~~~	61_45_4A	61_45_4A_1	0.014	ACSR16	1.268	0.422
Line29~	61_5	61	0.5	ACSR95	0.215	0.334
Line87	61_5	61_6	0.1	ACSR95	0.215	0.334
Line70~~	61_6_6A	61_6	0.648	ACSR16	1.268	0.422
Line88	61_7	61_6	0.1	ACSR95	0.215	0.334
Line90	61_7	61_7_2	0.18	ACSR16	1.268	0.422
Line100	61_7_2	61_7_8	0.524	ACSR16	1.268	0.422
Line93	61_7_2	61_7_2_1A	0.169	ACSR16	1.268	0.422
Line94	61_7_2_1A	61_7_2_1A_2	0.22	ACSR16	1.268	0.422
Line98	61_7_2_1A	61_7_2_2	0.014	ACSR16	1.268	0.422
Line24~	74_1A_4	74_1A_4_6	0.507	ACSR16	1.268	0.422
Line27	74_1A_4	S_FBI_288	0.008	ACSR35	1.071	0.393
Line28	87_3	S_FBA_210	0.01	ACSR35	1.071	0.393
Line27~~	87_3_1	S_FBSB_168	0.01	ACSR35	1.071	0.393
Line29	87_3_1	87_3	0.01	ACSR35	1.071	0.393
Line45~	92_13	92_8	0.451	ACSR35	1.071	0.393
Line62~	92_13	92_14	0.043	ACSR35	1.071	0.393
Line60~	92_14	92_18	0.479	ACSR35	1.071	0.393
Line33	92_18A	92_18A_7	0.571	ACSR35	1.071	0.393
Line63~	92_18A	92_18	0.01	ACSR35	1.071	0.393
Line37	92_18A_10	92_18A_26	1.384	ACSR35	1.071	0.393
Line39	92_18A_10	S_FBI_13	0.022	ACSR35	1.071	0.393
Line64~	92_18A_26	S_JASPER_K	0.075	ACSR35	1.071	0.393
Line36	92_18A_7	92_18A_10	0.244	ACSR35	1.071	0.393
Line67~	92_21	92_18A	0.266	ACSR16	1.268	0.422
Line76~	92_21	92_21_4	0.4	ACSR16	1.268	0.422
Line77~	92_21	92_21A	0.06	ACSR16	1.268	0.422
Line75~	92_21_10	92_21_66	4.968	ACSR16	1.268	0.422
Line73~	92_21_4	92_21_10	0.6	ACSR16	1.268	0.422
Line69~	92_21_4_3	92_21_4	0.228	ACSR16	1.268	0.422
Line84~	92_21A	92_30	0.359	ACSR16	1.268	0.422
Line24~~~~	92_30	92_30_1	0.007	ACSR16	1.268	0.422
Line23~~	92_40	92_30	0.368	ACSR16	1.268	0.422
Line55~	92_8	92_8_1	0.018	ACSR16	1.268	0.422
Line1	p1	p2	0.001	ACSR95	0.215	0.334
Line92	S_2~	61_7_2_1A_2	0.07	ACSR35	1.071	0.393
Line91	S_2~~	61_7_2_1A_2	0.198	ACSR35	1.071	0.393
Line127	S_FB_112	61_32_31_1	0.001	ACSR35	1.071	0.393
Line35	S_FBI_11	92_18A_7	0.006	ACSR35	1.071	0.393
Line25~~~	S_FBI_263~~	92_30_1	0.01	ACSR35	1.071	0.393
Line30	S_FBI_59	92_13	0.487	ACSR35	1.071	0.393
Line21~	S_FBI_67	39_51_3	0.14	ACSR35	1.071	0.393
Line14	S_FBI_70	61_15A_6	0.298	ACSR35	1.071	0.393
Line13	S_FBI_71	61_15A_6	0.001	ACSR35	1.071	0.393
Line31	S_FBP_15	92_14	0.125	ACSR35	1.071	0.393
Line10	S_FBSB_178~	61_11	0.277	ACSR35	1.071	0.393
Line32	S_FBSB_178~~	92_18	0.356	ACSR35	1.071	0.393
Line65~	S_JASPER_A	92_18A_26	1.297	ACSR35	1.071	0.393

### 1.3 Loads and generations

This line serves forty-five (45) medium to low voltage transformers that mostly serve the loads. Their connection points, their installed capacity and the maximum, minimum observed active and reactive loads are provided at Table 2.

**Table 2:** Connection point of line transformers and their characteristics.

*Node*	*Installed load capacity* (kVA)	*Maximum observed load (active power)* (kW)	*Maximum observed load reactive power)* (kVA)	*Minimum observed load (active power)* (kW)	*Minimum observed load reactive power)* (kVA)
28	50	17	10	1	1
31	485	164	102	12	7
39	720	243	151	17	11
45	100	34	21	2	1
49	320	108	67	8	5
61	920	311	193	22	14
74	150	51	31	4	2
80	660	223	138	16	10
89	75	25	16	2	1
91	100	34	21	2	1
92	250	85	52	6	4
39_18	1490	504	312	36	22
39_51	100	34	21	2	1
39_51_3	50	17	10	1	1
39_94	360	122	75	9	5
45_16A	810	274	170	20	12
45_1A_4	100	34	21	2	1
49_9	410	139	86	10	6
61_10_6	100	34	21	2	1
61_28	100	34	21	2	1
61_28_9	160	54	34	4	2
61_32_16	50	17	10	1	1
61_32_162	545	184	114	13	8
61_32_31	200	68	42	5	3
61_32_31_1	100	34	21	2	1
61_45	210	71	44	5	3
61_45_12	50	17	10	1	1
61_45_4A_1	100	34	21	2	1
61_5	75	25	16	2	1
61_6_6A	560	189	117	14	8
61_7_2	250	85	52	6	4
61_7_2_1A_2	410	139	86	10	6
61_7_2_2	250	85	52	6	4
61_7_8	175	59	37	4	3
68_3	160	54	34	4	2
74_1A_4	100	34	21	2	1
74_1A_4_6	160	54	34	4	2
87_3	160	54	34	4	2
87_3_1	250	85	52	6	4
92_21_4_3	160	54	34	4	2
92_21_66	250	85	52	6	4
92_21A	100	34	21	2	1
92_30_1	160	54	34	4	2
92_40	160	54	34	4	2
92_8_1	50	17	10	1	1

The renewable energy sources plants are connected to the nodes as depicted to Table 3. They have mostly installed capacity of one-hundred kilo-watt (100 kW). However, on this line they connected larger plants up to 1,8 MW as well as, there are smaller installations of 20 kW. The production for these plants shall be considered to the average photovoltaic production in Greece.

**Table 3:** Photovoltaic plants connection points and their installed capacity.

*Node*	*Installed power (kW)*
S_FBI_67	100
S_FBI_288	100
S_FBA_210	100
S_FBSB_168	100
S_FBP_14	500
S_FBA_259	100
S_2~	100
S_2~~	100
S_FBSB_178~	100
S_FBI_70	100
S_FBI_71	100
S_FBI_69	100
S_FB_112	100
S_FBI_137	100
S_HBSE_470	470
S_HBSE_700	700
S_FBI_59	100
S_FBP_15	500
S_FBSB_178~~	20
S_FBI_11	100
S_FBI_13	100
S_JASPER_K	1274
S_JASPER_A	1815
S_FBI_263~~	50

## 2 Experimental design, materials and methods

The line is constructed using Aluminium Conductors Steel Reinforced (ACSR) of 16 mm^2^, 35 mm^2^ and 95 mm^2^. Their electrical characteristics are provided to Table 4. The accuracy of the simulations was decided to three decimal places.

**Table 4:** Resistance and reactance for the applicable to this dataset type of lines.

	R (Ω/km)	L (Ω/km)
ACSR16	1.268	0.422
ACSR35	1.071	0.393
ACSR95	0.215	0.334

To perform load flow analysis on Matpower, we have chosen per unit system. The base power is 10 MVA since this corresponds safely to the operation margin of the line. The base voltage is of 20 kV, which is the typical voltage level for medium voltage distribution lines in the country where this line operates. This decision for the base power and voltage leads to a base impedance of 4 Ω. Consequently, all resistances and reactances and expressed in per unit and the length is taken into consideration. The calculations are available on the excel file available to the data repository (Table 5).

**Table 5:** Per Unit values applied to this simulation.

	Base power	Base voltage	Base impedance
p.u.	10 MVA	20 kV	4 Ω
